# IQSeq: Integrated Isoform Quantification Analysis Based on Next-Generation Sequencing

**DOI:** 10.1371/journal.pone.0029175

**Published:** 2012-01-06

**Authors:** Jiang Du, Jing Leng, Lukas Habegger, Andrea Sboner, Drew McDermott, Mark Gerstein

**Affiliations:** 1 Department of Computer Science, Yale University, New Haven, Connecticut, United States of America; 2 Program in Computational Biology and Bioinformatics, Yale University, New Haven, Connecticut, United States of America; 3 Department of Molecular Biophysics and Biochemistry, Yale University, New Haven, Connecticut, United States of America; University of Chicago, United States of America

## Abstract

With the recent advances in high-throughput RNA sequencing (RNA-Seq), biologists are able to measure transcription with unprecedented precision. One problem that can now be tackled is that of isoform quantification: here one tries to reconstruct the abundances of isoforms of a gene. We have developed a statistical solution for this problem, based on analyzing a set of RNA-Seq reads, and a practical implementation, available from archive.gersteinlab.org/proj/rnaseq/IQSeq, in a tool we call IQSeq (Isoform Quantification in next-generation Sequencing). Here, we present theoretical results which IQSeq is based on, and then use both simulated and real datasets to illustrate various applications of the tool. In order to measure the accuracy of an isoform-quantification result, one would try to estimate the average variance of the estimated isoform abundances for each gene (based on resampling the RNA-seq reads), and IQSeq has a particularly fast algorithm (based on the Fisher Information Matrix) for calculating this, achieving a speedup of 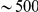 times compared to brute-force resampling. IQSeq also calculates an information theoretic measure of overall transcriptome complexity to describe isoform abundance for a whole experiment. IQSeq has many features that are particularly useful in RNA-Seq experimental design, allowing one to optimally model the integration of different sequencing technologies in a cost-effective way. In particular, the IQSeq formalism integrates the analysis of different sample (i.e. read) sets generated from different technologies within the same statistical framework. It also supports a generalized statistical partial-sample-generation function to model the sequencing process. This allows one to have a modular, “plugin-able” read-generation function to support the particularities of the many evolving sequencing technologies.

## Introduction

The concepts of genes and isoforms have evolved and become more complex [1]: the discovery of splicing [2]–[4] revealed that the gene was a series of exons, coding for, in some cases, discrete protein domains, and separated by long noncoding stretches called introns. With alternative splicing, one genetic locus could code for multiple different mRNA transcripts (isoform transcripts). This discovery complicated the concept of the gene radically. For instance, as of 2007, the GENCODE annotation [5] contained on average 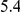 transcripts per locus.

With the recent development of high-throughput RNA sequencing (RNA-Seq) technology, it is possible for biologists to measure transcription with unprecedented precision. One problem that can now be tackled is that of isoform quantification, where one tries to reconstruct the abundances of similar isoforms based on a set of RNA-Seq reads. Various methods have been developed to solve this problem. In previous work, researchers proposed different statistical frameworks to solve this problem. Xing et al. [6] proposed a maximum likelihood problem, an expectation maximization solution, and a Fisher information measurement for performance estimation; Jiang et al. [7], based on Poisson model assumption, formulated a maximum likelihood problem and its numerical solution, and also utilized the observed Fisher information matrix to sample the posterior distribution of isoform quantity; Trapnell et al. [8] used variable read-length model (normal distribution by default) and a sampling method similar to [7] to derive the posterior distribution of isoform quantity; Richard et al. [9] with a Poisson model, also used bootstrapping to study the robustness of their method against non-uniform sequencing effects; Lacroix et al. [10] studied the conditions under which the problem can be solved, revealing that although neither single nor paired-end sequencing guarantee a unique solution, paired-end reads may be sufficient to solve the vast majority of the transcript variants in practice.

These studies, however, have not fully addressed the problem of isoform quantification in a couple of respects: First of all, they usually assume that only one sequencing technique is used in an experiment, and that the reads are uniformly sampled along the transcripts. These are not necessarily good approximations to real data. Second, while some theoretical results have been presented on estimating the accuracy (e.g. average variance) of quantification results, there does not yet exist a method to efficiently compute these measurements other than using brute-force simulation, which is computationally infeasible in large scale expriments involving tens of thousands of genes and millions of sequencing reads. On the other hand, fast estimation of quantification accuracy would not only enable researchers to better understand the analysis results being obtained, but also will be useful in RNA-Seq experiment design to optimally integrate different sequencing technologies in a cost-efficient way.

In order to fill in these gaps, we have developed a generalized statistical solution for the problem of isoform quantification, and a practical implementation in a tool we call IQSeq (Isoform Quantification in next-generation SEQuencing). IQSeq has the following features which represent improvements over previous work in isoform quantification in the following aspects:

1. It has a generalized statistical read generation function during the sequencing process (i.e. a customizable function describing how reads are randomly sampled from isoforms). This provide a flexible way to incorporate characteristics of different sequencing technologies (e.g. 3′ end sequencing bias of transcripts).
2. It integrates the analysis of different sample sets generated from different sampling technologies (e.g. long and short reads).
3. It has a fast algorithm for estimating the average variance of the results provided by our expectation maximization based solution.
4. Given the estimated isoform abundance output, IQSeq also provides an information theoretical method to measure the overall transcriptome complexity.

In this paper, we will first introduce a mathematical definition of the generalized partial sampling and distribution estimation problem (which IQSeq is based on), and provide a expectation maximization based iterative solution. Then we discuss in detail on how to estimate the performance of this solution using Fisher information based heuristics, and present fast algorithms that implement the computation of these heuristics. Finally, we show results of applying our methods to both simulated and real-world data, illustrating scenarios where such integrated analysis can be the most informative.

## Methods

First, we formally define the isoform quantification with multiple sequencing technologies as a generalized statistical partial sampling problem, and present a computational solution based on maximum likelihood estimation and expectation maximization. We then show both analytical results and practical fast algorithms to estimate the average variance of the solution on isoform quantification, and compare their computational complexity against brute-force methods. We present the main theoretical results in this section, and detailed derivations can be found in Text S1.

### Problem Definition

We start by defining the generalized process of batch partial sampling, which represents the sequencing process in RNA-Seq experiments, and the relationships between partial samples and the objects being sampled.

#### Definition 1

(Batch Partial Sampling) Let 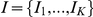 be all the possible isoforms for a given gene, with relative abundances 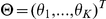, where 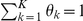. We assume that there are 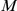 different partial sampling methods (sequencing techniques with difference characteristics, e.g. long/medium/short, single/paired end): 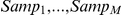, and let 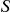 denote all the samples (reads): 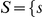 from 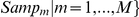. We also define 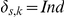 (partial sample (read) 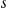 is compatible with 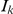), where 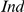 is the indicator function. There are in total 
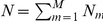
 samples, where 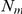 is the total number of partial samples from 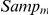.

Here we assume a two-step sampling process: First, a sampling method 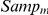 chooses an isoform instance 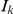 according to 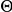. Second, the sampling method generates a partial sample 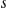 according to a local partial sample generation model (the read generation function) 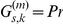 (generating 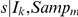).

#### Definition 2

(Distribution Estimation based on Batch Partial Samples) *Given* 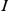, and 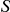 as defined in Definition 1, estimate 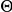.

**Figure 1:**
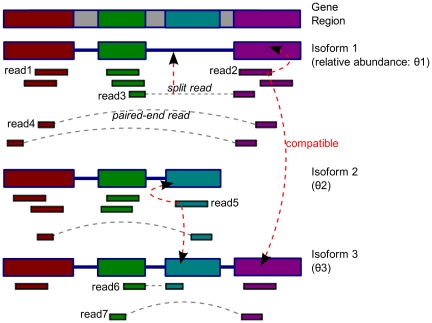
Reads (partial samples) in the isoform quantification problem.

As shown in Figure 1, 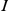 are the isoforms with different relative abundances 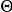, and 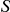 are the single- and paired-end reads whose sequences align with part of this gene region. Some of these reads (e.g. read 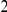, 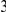 and 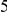) are compatible with multiple isoforms. The ultimate problem is to estimate 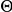 based on 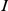 and 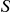, i.e., reconstructing a distribution based on partial observations.

In the remaining part of this paper, we will use two notations to describe a partial sample 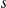: 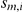 is the 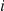
th sample from 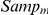; and 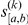 stands for a partial sample from 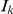, starting (inclusive) from position 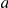 and ending (exclusive) at 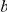 in that isoform. We also define exons as those nodes in the splicing graph of a gene, so that there are no exons that overlap with each other (i.e. an exon in a transcript may be a combination of multiple nodes of the splicing graph). We have included in our software package a preprocessing tool for grouping transcripts into gene clusters and formulating corresponding splicing graphs.

### Maximum Likelihood Estimation (MLE)

Definition 2 does not give an explicit criterion for a “good” estimation of 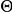. Since the problem is defined in a statistical sampling framework, it is natural to consider using Maximum Likelihood as such a criterion.

#### Definition 3

(Maximum-Likelihood Distribution Estimation based on Batch Partial Samples) Given 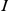, and 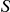 as defined in Definition 1, find 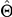 such that:
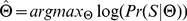
(1)


By plugging in the partial samples 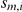
s and 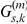
s, we can rewrite the formula above as follows:
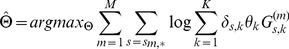
(2)


In the next subsection, we demonstrate how this problem can be solved by introducing a hidden variable 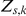 and using the technique of Expectation Maximization [11].

### Applying the Expectation Maximization Method

We define 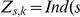 is from 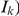, which are the hidden variables in this problem. Since Expectation Maximization gives an iterative solution, we denote the estimation for 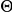 in the 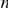
th step as 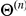, and further define 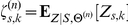, which is the expectation of 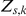 given 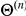 (the estimated paramters at the 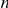
th step) and the reads 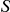.
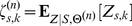
(3)

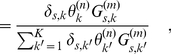
(4)where 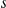 is generated by 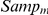.

By performing an E step that computes
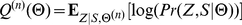
(5)

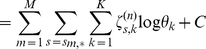
(6)and a M step which maximizes 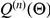 with constraint: 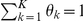, we have:
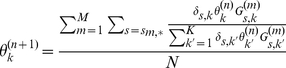
(7)as the new estimation for 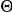.

The iterative estimation in Equation 7 is intuitively consistent with the case of estimating a distribution based on full samples: consider the scenario in which for each 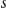, there is only one 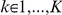 satisfying 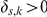, the right hand side of Equation 7 thus becomes 
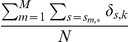
, which is exactly how the distribution estimation problem with traditional full samples can be solved. In the case of partial samples, our solution provides a way to adjust the “weight” each sample 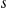 contributes to the 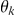
s of different objects.

### Analyzing the Performance of Estimation

Given 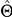 obtained from the MLE solution presented in the previous section, we would like to understand how much this estimate will deviate from the “true” 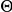 on average. Here we focus on the variance of the 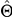, which describes how stable the MLE result is over many different partial sample sets (obtained via additional experiments or re-sampling) drawn from the same isoform set:
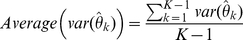
(8)


As we will show later, although brute-force simulation can be performed to obtain a relatively accurate estimation of this measurement, it is may become computationally intractable when there are too many reads and genes to be considered. We thus propose to use a Fisher information based heuristic for estimating 
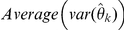
, and present a fast algorithm to compute the exact value of this heuristic.

We first introduce the Fisher information matrix [12], [13] as a basis for further discussion. The Fisher information is a way of measuring the amount of information that the random samples 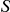 carries about the unknown parameter 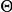 upon which the likelihood function of 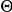, 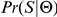, depends. An important use of the Fisher information matrix in statistical analyses is its contribution to the calculation of the covariance matrices of estimates of parameters fitted by maximium likelihood.

Let 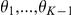 be the free parameters, and 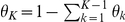.

#### Definition 4

(Observed Fisher information matrix).
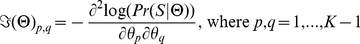
(9)

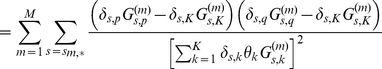
(10)


#### Definition 5

(Expected Fisher information matrix).
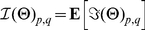
(11)


### Covariance matrix of the maximum likelihood estimator

Let 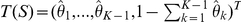, and 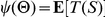. The Cramér-Rao bound [13] states that:
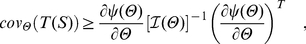
(12)where 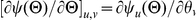, 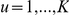; 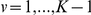.

We then estimate 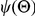 by 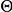, and use the bound above to estimate the covariance matrix:
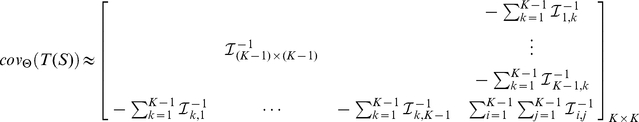
(13)


This means that we only need 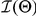 in order to estimate the performance of our MLE with different sampling method combinations.

### Heuristic for MLE performance estimation

In order to provide a single value measure for the expected performance of Maximum Likelihood estimation, we propose to use the following heuristic to estimate the average variance of 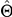:
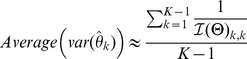
(14)


This heuristic avoids the potential computational intensive and numerically unstable computation of the inverse of 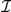, and is consistent with the theoretical result on the lower-bound of 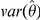 in one dimensional case:
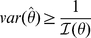
(15)which is a specialization of the result in the previous subsection. In other words, the precision to which we can estimate 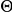 is fundamentally limited by the Fisher information.

In order to compute this heuristic, all we need is 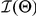 itself. However, the brute-force computation (according to Definition 4 and 5) of this matrix will be time-consuming since its time complexity is proportional to the total number of possible sample sets (which in turn grows exponentially with the number of samples). In the next section, we will present algorithms that can compute this matrix in a more efficient fashion.

### Efficient Computation of 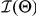


First of all, we can decompose 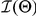 in the following way:
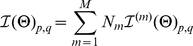
(16)where
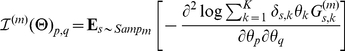
(17)is the expected Fisher information matrix of a single partial sample based on 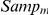. Thus we need to be able to compute 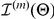 in order to obtain 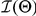.

### Further decomposing 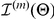




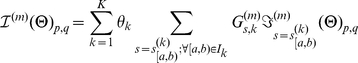
(18)where
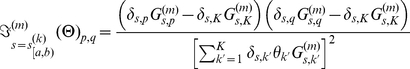
(19)is the Fisher information matrix of a partial sample 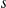 from 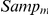 at 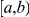 in 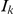.

A brute-force algorithm for computing 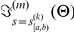 can thus be described as follows: Algorithm 1

**Figure 2:**
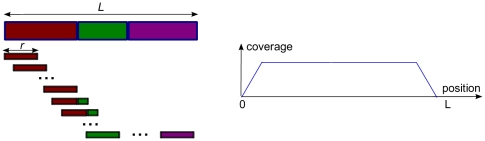
A simple shotgun read generation model.

In Algorithm 4, if 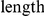 is the length of a given sequence 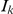, then the whole algorithm consists of 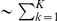 length(*I* *_k_*) computations of 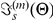.

### Equivalent partial samples

In order to continue our discussion on faster algorithms to compute 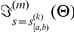, we introduce the concept of equivalent partial samples below (the relavant proofs can be found in Text S1):

#### Definition 6

Two partial samples 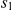 and 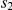 are equivalent w.r.t. 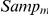 if and only if 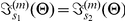.

#### Lemma 1

*If* 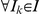 *,* 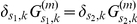 *, then* 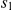 *and* 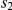 *are equivalent w.r.t.* 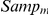 *.*

#### Definition 7

A set of partial samples 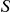 is an equivalent sample set w.r.t. 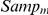 if and only if 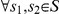, 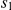 and 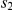 are equivalent w.r.t. 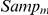.

#### Lemma 2

*Given an isoform* 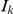 *and a sampling method* 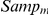 *, if we divide all its possible partial samples into* 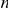 *non-overlapping equivalent sample sets* 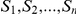, *then:*

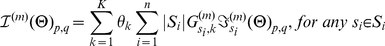
(20)


### Results from a simple shotgun read generation model

In this subsection, we consider a simplified partial sample generation model:

#### Definition 8

A simple shotgun sampling method 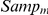 generates samples with fixed read length 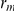. When sampling from an isoform 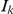 with length 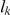, there are in total 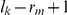 different samples 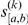, where 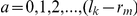; and 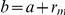. Each of these samples has equal probability of being generated from 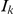: 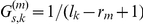.

Figure 2 illustrates simple shotgun sampling process and its corresponding per-base coverage on the isoform being sampled.

**Figure 3:**
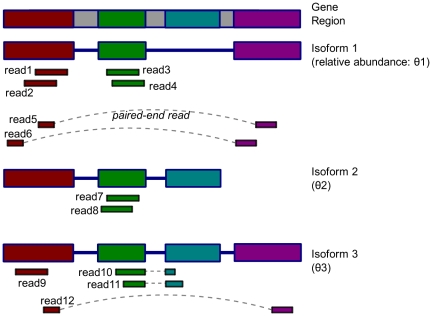
Equivalent samples in a simple shotgun read generation model.

#### Lemma 3

*Given the sample generation model* 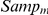 *above, if two samples* 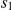 *and* 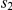 *generated by this method are compatible with the same set of isoforms, i.e.* 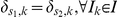 *, then* 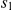 *and* 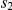 *are equivalent w.r.t.* 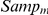 *.*

#### Theorem 1

*Given the sample generation model* 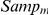 *above, if two samples* 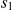 *and* 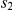 *generated by this method overlap with all the junctions in the same set of connected exons* 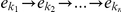 *, then* 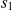 *and* 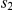 *are equivalent w.r.t.* 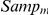 *.*

For example, in Figure 3, where the reads are generated from a simple shotgun sampling process, the equivalent partial samples are -read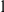, read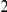, read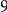}, -read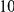, read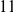}. Also, if we consider a paired-end read as a long shotgun read with its gap filled, the samples read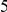 and read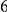 are also (approximately) equivalent, if their insert sizes are close to each other. However, read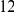 is not equivalent to these reads, since its shotgun version overlaps with a different exon junction set (with an addition exon).

**Figure 4:**
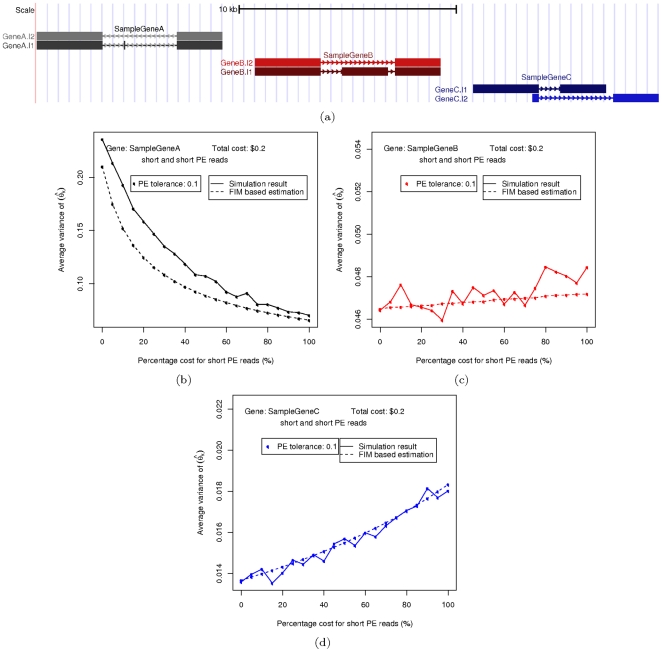
Results on simplified genes. (a) Results on gene A (b) Results on gene B (c) Results on gene C.

### Algorithms for efficiently computing 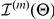


Based on Definition 8 and Theorem 1, we can design the following algorithm for efficiently computing 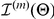 by combining the computation of this value for equivalent partial samples from each isoform. Algorithm 2

**Figure 5:**
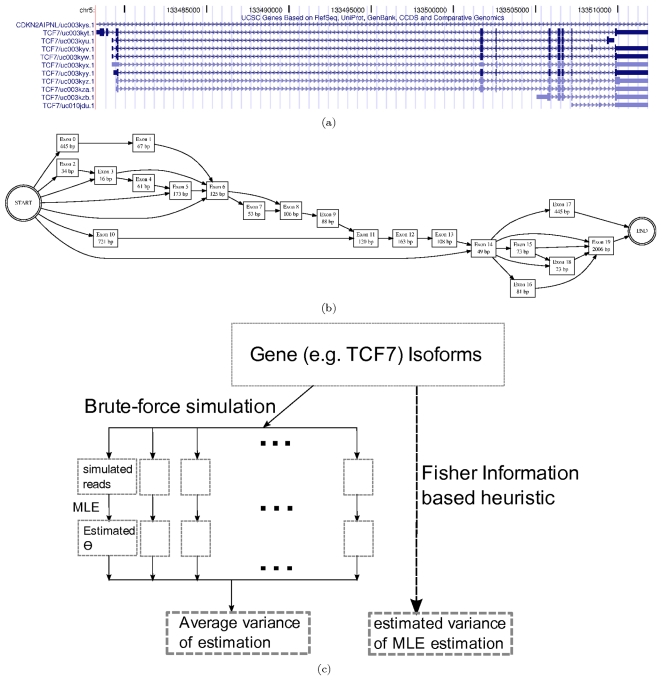
Computations on gene TCF7. (a) Known isoforms (b) Splicing graph (c) Simulation schema.

In Algorithm 2, 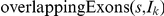 identifies the connected exons set in 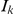 that overlaps with a given partial sample 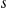, and can be implemented with 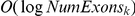 time complexity by pre-computing an exon-position index table for the isoforms.

We can further reduce the number of times of computing 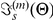 by combining equivalent partial samples from across isoforms: when an equivalent sample set from an isoform has been identified, all the same samples from other isoforms can be recorded in lists of intervals to avoid redundant computation of their 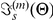
s. The algorithm is shown below: Algorithm 3

**Figure 6:**
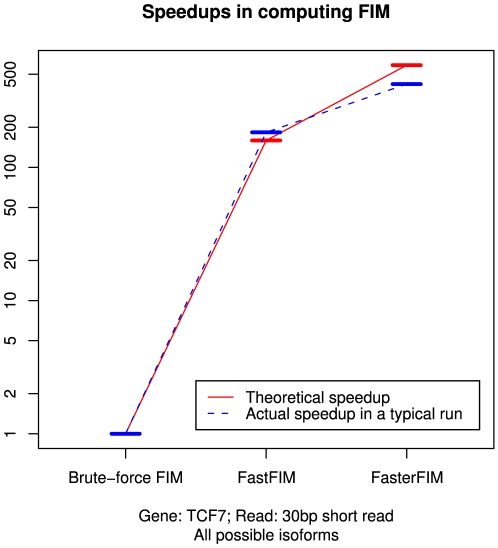
Speedup in FIM computation for gene TCF7.

In Algorithm 3, 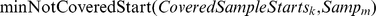 finds the minimum position 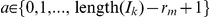 that is outside a given interval list 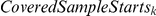; 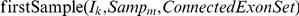 returns the partial sample 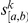 from 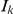 covering all the exon junctions in 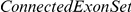 with a minimum 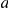, and can be implemented with a worst-case 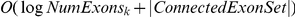 time complexity by using a pre-computed exon position index table for the isoforms.

### Complexity analysis

Given a set of 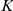 possible isoforms 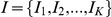, with lengths 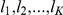, respectively, and a shotgun sampling method 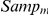 with sample read length 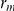 as described in Definition 8, Algorithm 1 requires 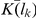 steps of computing 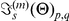. Thus computing 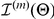 using this brute-force algorithm requires 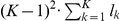 operations of calculating 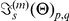. If we assume that the average length of an isoform is 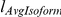, this corresponds to 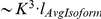 computations of 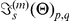.

Suppose that on average an isoform can be divided into 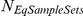 equivalent sample sets by Algorithm 2, this algorithm will then require 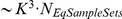 steps of computing 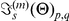 to obtain the Fisher information matrix 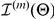 for the given sampling method, thus being more efficient than Algorithm 1 by a ratio of 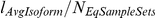. Algorithm 3 will obviously be even more efficient by avoiding the redundant computation of some of the equivalent sample sets in Algorithm 2.

### Using more complex 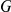 function in Algorithm 3

The sequencing technology being used in an RNA-Seq experiment is usually more complicated than the simplified 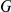 function described in Definition 8, which assumes equal sample-length and uniform generative probability. In reality, a typical 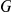 usually involves reads with different lengths within a certain range, and also biased sample generation probability at different locations of a full-length isoform. Although once such a 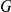 is defined, our MLE solution can treat it in the same way as it does for simplified versions, Algorithm 3 no longer works “out of the box” due to its dependency on Definition 8 to find equivalent partial samples. We discuss briefly in this subsection on how to handle more complex features.

When the assumption of uniform sample generation still holds, it is straightforward to handle samples with different lengths in FIM computation. We can treat one sampling method as a combination of multiple simplified methods as in Definition 8, with different sample lengths 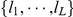:
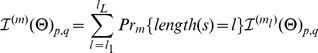
(21)

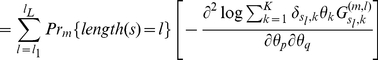
(22)where 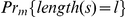 represents the probability of generating a sample with length 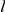 in sampling method 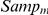, 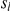 is a sample with length 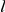, and 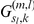 is the simplified sample generation probability as in Definition 8, with sample length 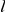.

In the case of non-uniform sample generation along the isoform, if 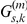 is a step function (piece-wise constant function) for sample 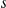 along isoform 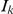, we will still be able to find equivalent sample sets as described in Definition 7, based on both the isoform structures and the intervals in 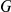. If, however, very few such constant components exist in 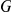, we will need to relax the definition of equivalent partial samples to satisfying 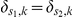 only. With this relaxed definition, we can find samples 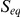 with equivalent structural similarities to all the isoforms. In this case, if the isoforms contain regions where any 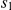 and 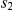 from it satisfy 
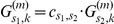
 with a constant 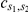 for all 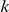, we still have 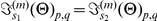 according to Equation 19, and the 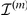 can thus be efficiently computed using a variant of Algorithm 3 by combining the computation for such equivalent partial samples. For more complex 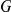 functions, however, approximation algorithms may have to be introduced for fast computation of 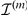.

## Results

### Simulation Results

Here we present our results on a set of simulated datasets. In order to demonstrate the accuracy and efficiency of the methods we developed, we first use simulations to show the performance of our approach on simplified gene models and a real gene. These simulations are useful in designing optimal sequencing experiments for isoform quantification.

### Simulation on simplified genes

Due to the complexity of real gene structure, we apply our methods to three artificially constructed genes with simplified isoform structures, so as to better illustrate how different characteristics of the gene structures can affect the outcome of the isoform quantification analysis.

As shown in Figure 4A, each of these genes has two different isoforms, with the more abundant one shown in a darker color. Two sampling techniques, short single and short paired-end (PE), are used to generate reads from them, with a fixed total cost of $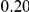 (roughly corresponding to 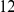 medium length reads with average size of 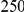 bp (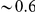
× coverage on the simplied genes), or 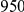 short reads with average length of 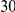 bp (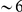
x)). The per-base costs of these sampling techniques are based on [14]. Different cost combinations, e.g. different percentage of the total cost being assigned to a certain sampling technique, are illustrated by the 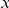-axis in Figure 4B–D. For each of these cost combinations, we randomly generate 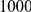 read sets, and use the MLE solution to estimate 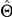, based on which 
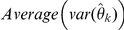
 are computed (solid lines in Figure 4B–D). We also use Algorithm 3 to estimate the same quantity, and plot the estimations using dashed lines in the same figure for comparison. The results show that the FIM estimation of 
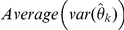
 are close to the direct simulation results, and also correctly predicts the trend in how this value changes with different cost combinations. Also, different gene structures have noticeable impact on the MLE accuracy, mostly due to the ability of sampling techniques to distinguish isoforms from each other with different gene structures.

**Figure 7:**
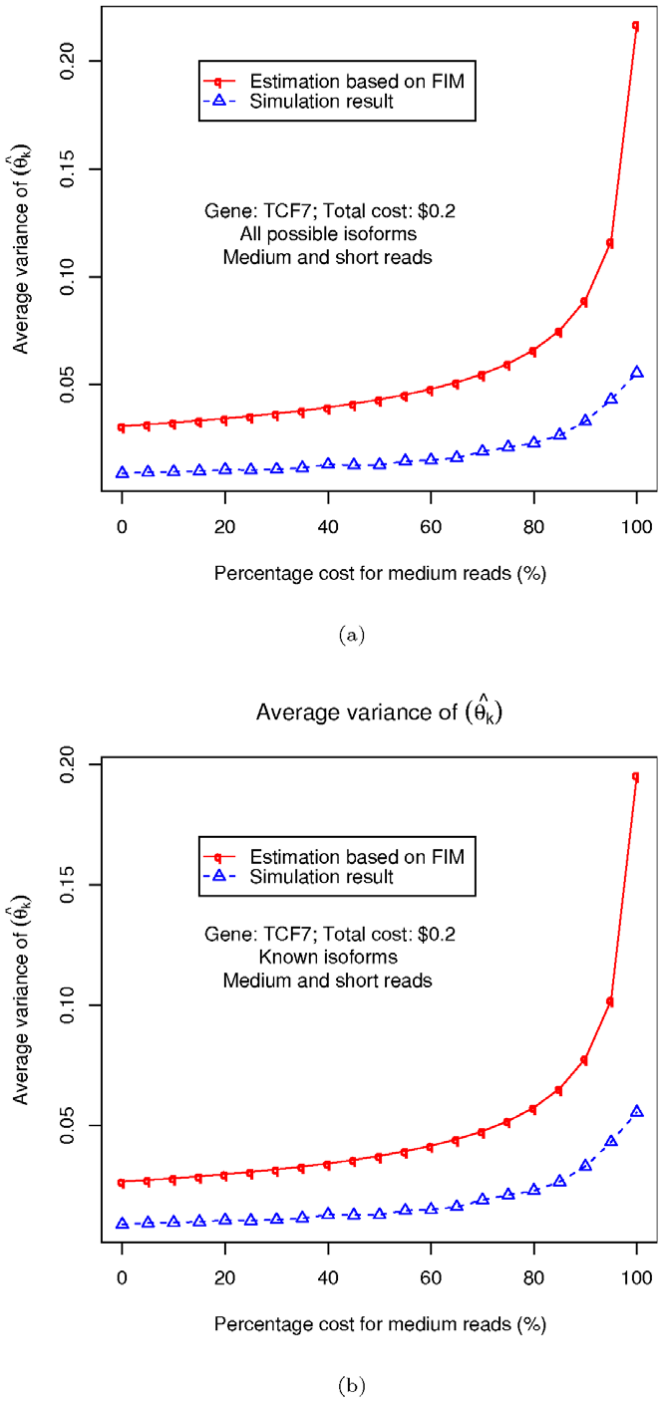
Simulation results on TCF7. (a) Results on all possible isoforms (b) Results on known isoforms.

Not only can the FIM based heuristic correctly approximate how the performance of MLE changes with regard to different sampling technique combinations, it is also able to dramatically shorten the time of computation, as shown in Table 1. This is mainly because while the computation of brute-force simulation depends heavily on the number of reads being generated and the number of trials needed to obtain a relatively stable estimation of 
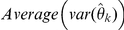
, the core computation taken by the FIM based heuristic is the evaluation of individual FIMs for the sampling techniques involved, which can be efficiently computed using Algorithm 3, and then combined based on Equation 16 to estimate 
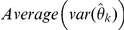
 under different cost combinations. Being able to do these simulations fast is useful in designing optimal expriments.

**Table 1:** Total time used by brute-force simulation vs. FIM based heuristic to estimate 
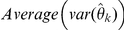
 in simplified genes.

**Total trials for one gene**	Number of trials 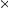 Number of sampling method combinations 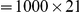
**Total FIM computation for one gene**	Number of sampling methods 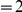
**Total CPU time used by brute-force simulation**	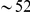 minutes
**Total CPU time used by FIM based heuristic**	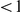 second

### Efficiently Estimating quantification error: Application on a typical gene

We have developed a Fisher information matrix (FIM) based fast algorithm (Algorithm 3 in Methods section) for estimating the quantification error in 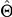, and compared its speed with two other benchmark algorithms. Here we consider the gene 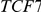, which has 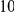 known isoforms shown in Figure 5A. Figure 5B shows its corresponding splicing graph [6], [15], with 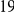 exon blocks, and 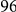 possible isoforms, which are all the possible paths from node “START” to node “END” in the splicing graph. Figure 5C shows the brute-force way and Fisher information based method to estimate 
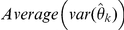
.

**Figure 8:**
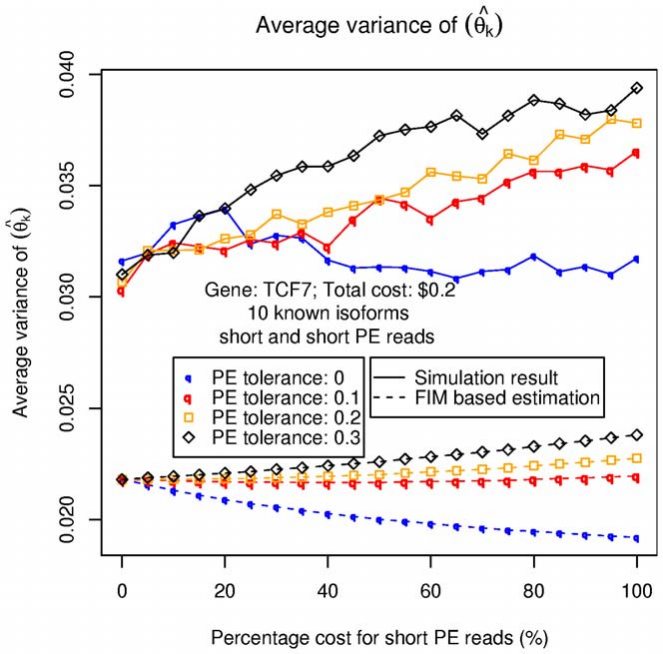
Simulation results on TCF7 with paired-end reads.

When computing the expected Fisher information matrix 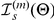, a brute-force algorithm (Algorithm 1) requires 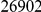 computations of the observed Fisher information matrix 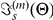, while an improved algorithm developed by us (Algorithm 2) involves 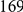 such computations, and the number for our final algorithm (Algorithm 3) is 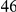, achieving a 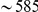 times speedup compared to the brute-force method. A summary of the speedups is shown in Figure 6. Note that theoretical speedup is calculated based on the number of key computational steps (per-read FIM computation), while the actual speedup depends on the software implementation of all steps in the algorithm.

**Figure 9:**
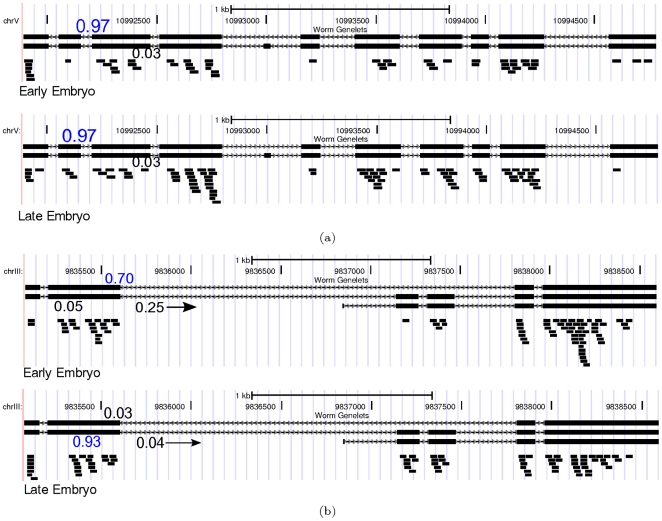
Isoform composition in two stages. (a) Gene 14047 in two stages ( *Diff* 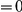) (b) Gene 7649 in two stages ( *Diff* 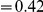).

### Integrated analysis with multiple sequencing technologies: Simulation on a typical gene

We present in this section the application of the FIM based heuristic on a real gene, and compared the results to the ones obtained from direct simulations. We pick TCF7 again as a typical example gene with multiple isoforms. Similarly to our procedure in the section on simplified genes, two sampling techniques, medium and short shotgun sequencing (with average length of 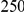 bp and 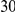 bp, $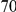 and $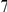 per 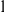 million base cost respectively), are used to generate reads from them, with a fixed total cost of $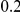, with 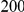 trials being conducted for each cost combination. Two different sets of results are shown in Figure 7, one using all the 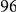 possible isoforms deduced from its splicing graph, and the other just using its 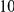 known isoforms. As in the previous section, the results here show that the FIM estimation of 
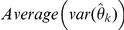
 are close to the direct simulation results, and also correctly predicts the trend in how this value changes with different cost combinations.

**Figure 10:**
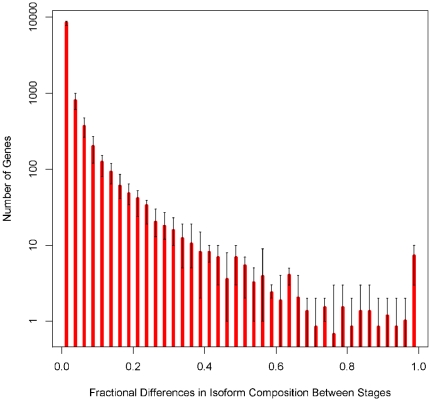
*Diff* of all genes across four stages.

Figure 8 presents a more detailed simulation focusing on short paired-end reads. The 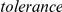 value reflects the expectation of the variance in insert size for such experiments: a 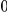 value means that all the paired-end reads are expected to have exactly the same insert size; the higher the 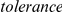 is, the more relaxed are we on the insert size variation (i.e. if the distance of the mapped positions of the two ends of a read on a transcript is within the expected insert size 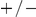 a “tolerated” ratio, the paired-end read will be considered “compatible” with the transcript). As we can see from this figure, the higher the 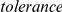, the larger 
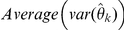
 becomes, corresponding to a worse expected performance of MLE. This can be explained by the fact that a higher 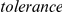 makes the sampling method less capable of distinguishing highly similar isoforms from each other based on a single paired-end read (e.g. GeneA in Figure 4A). The FIM based heuristic is again able to correctly depict the different trends of MLE performance under different cost combinations and 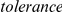 settings.

**Figure 11:**
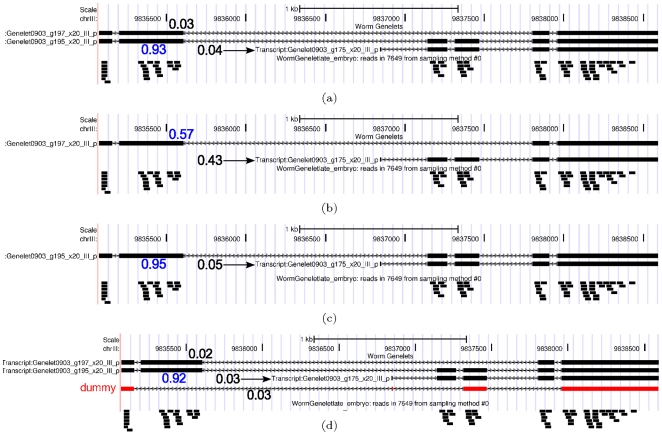
Gene 7649: Leave out one isoform, or add a “dummy” isoform. (a) Standard calculation with all isoforms: 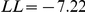 (b) Leave out the dominant isoform: 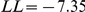 (c) Leave out a non-dominant isoform: 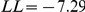 (d) Add a “dummy” isoform: 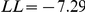.

We also show the computation time used by brute-force simulation and FIM based heuristic in Table 2. Note that the brute-force simulation is even more computational consuming, mainly because more isoforms are involved in the MLE process. Given the fact that there exist more than 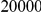 genes in the human genome and that the simulation has to be rerun for every new experiment to adjust its read counts (the number of reads attributed to a gene region in the experiment), using the FIM based heuristic instead for the purpose of estimating isoform quantification accuracy is obviously a more computationally tractable choice.

**Table 2:** Total time used by brute-force simulation vs. FIM based heuristic to estimate 
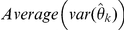
 in TCF7.

**Total trials for one gene**	Number of trials 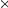 Number of sampling method combinations 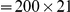
**Total FIM computation for one gene**	Number of sampling methods 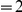
**Total CPU time used by brute-force simulation**	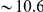 hours
**Total CPU time used by FIM based heuristic**	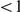 second

### Application to a model-organism (worm) dataset

To illustrate how we can interpret the 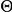 values output, we further apply our MLE solution to a worm dataset [16]–[18], which is a well-studied model organism. which is a well-studied model organism. The worm has intermediate complexity in isoform structures. It has isoforms but they are significantly simpler in structure than in human, leading to interpretability in the results. This dataset includes multiple developmental stages, and we were able to compare the results on a same set of isoforms under different conditions. The worm genome contains 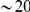 K genes, and the transcripts from each stage are sequenced with 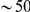 M short Solexa reads. This dataset is particularly useful for isoform comparison since it contains multiple stages of splicing events that are not overly complex.

### Dataset description

Whole transcriptome sequencing data for worm L2, L3, L4 and Young Adult stages, each stage with on average 50 M reads. The annotation set (derived from the modENCODE project, [17], [18]) has 21774 total genes. Of these, 12875 genes has multiple isoforms, with an average of 4.344 isoforms per gene.

### Comparison of isoform composition between stages

We first present the isoform quantification results on individual genes in two different stages, early embryo (EE) and late embryo (LE), to briefly illustrate the fact that different genes have different isoform composition differences between stages. Here we use the following formula to measure the difference in isoform composition of the same gene in two different stages:
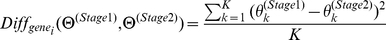
(23)where 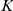 is the total number of isoforms in gene 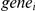.

Figure 9 shows two examples of zero and non-zero *Diff* values. The reads are plotted below the isoforms, and the numbers associated with the isoforms are their estimated relative abundances based on MLE. Furthermore, if we compute such values for all the genes in these two stages, we can get a histogram of isoform composition differences as illustrated in Figure 10, which characterizes the general isoform composition difference between stages. The distribution of differences in relative isoform composition for genes is shown: Isoform quantification was applied to RNA-Seq data in 4 developmental stages in worm (L2, L3, L4, YA) and *Diff* score was calculated for each gene in all 6 pairwise comparisons. Because isoform quantification is noisy for genes expressed at very low level, we plotted the distribution for genes that have at least an RPKM value of 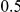 here (RPKM for a gene is the sum of RPKMs of all its isoforms). Red bars represent the average number of genes within the respective *Diff* score range, while error bars indicate the maximum and minimum numbers. *Diff* scores close to 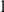 indicate big changes in isoform composition, or the relative expression level of isoforms between stages. The histogram indicates that only a few genes (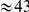) show dramatic differences in isoform expression between stages. (The number 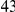 is derived from a cutoff of 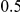 on the *Diff* score.) In Table 3, we include a classification of the structural difference (5′ UTR, 3′ UTR, alternative exon, etc.), between the dominant transcripts in such genes with different isoform compositions. When the different dominant isoforms from a gene differ in two aspects, we assign 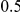 to each category. As shown in this table, many of the structural differences are due to either Distinct 5′ UTR or Overlapping 3′ UTR. We have also included in the supplementary website (http://archive.gersteinlab.org/proj/rnaseq/IQSeq) the genes with stage-wise isoform composition differences, ranked by their FIM based estimation variances, and with a thresholded on *Diff* score and RPKM at 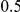.

**Figure 12:**
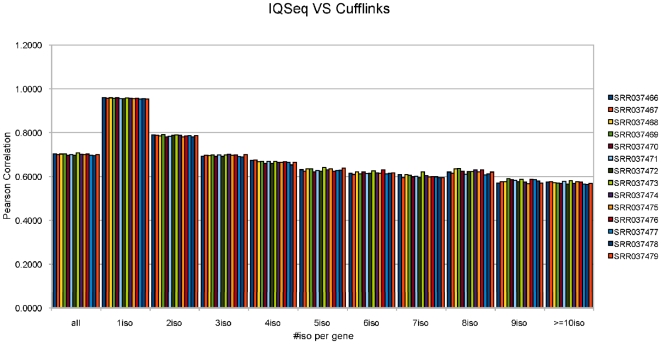
Comparison between IQSeq and Cufflinks.

**Algorithm 1:**
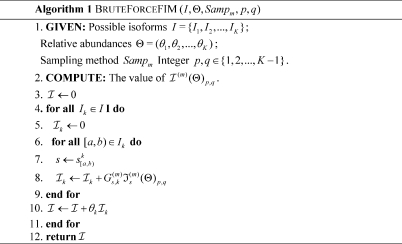
BruteForceFIM (*I,*Θ*,Samp_m_,p,q*)

**Table 3:** Classification of different isoform composition between stages.

Type	L3 vs L2	L4 vs L2	L4 vs L3	YA vs L2	YA vs L3	YA vs L4
**Overlapping 5′ UTR**	6.5	6	2.5	4	4.5	2.5
**Distinct 5′ UTR**	7.5	26	13.5	20	11	11
**Alternative Exon**	4.5	6.5	3	4.5	3	4.5
**Extended Exon**	4	7	6.5	6.5	6	6.5
**Overlapping 3′ UTR**	13.5	11	8.5	11.5	9.5	10
**Distinct 3′ UTR**	2	3.5	3	2.5	3	1.5

### The effect of different isoform sets on MLE result

We also investigate how different isoform sets (e.g. with a major/minor isoform missing, with an additional “dummy” isoform) will affect the MLE result, especially in terms of the maximized likelihood value. We pick gene No. 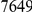 as a base isoform set, using the same set of reads and the per-read average maximized likelihood value 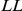 to measure the goodness of fitting:
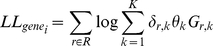
(24)


As we can see from Figure 11, the 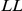 value always decreases when we modify the “true” isoform set in an unfavorable fashion. This shows that the likelihood score is an effective metric for ranking isoform sets for a particular gene. We observe that the 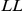 value decreases the most in all cases when the dominant isoform is removed from the isoform set Figure 11b), which indicates that a more important element has a larger contribution in explaining the generated reads. Correspondingly, when a low-probability or dummy isoform (that is not similar to the dominant one) is added to the input isoform set, the 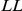 value decreases less significantly (about half of the case with dominant isoform removal), and also the isoform quantification results remain almost unchanged for the other transcripts in the isoform set. In practice, this characteristic can also be useful to eliminate non-existing isoforms - any isoform that has little effect on either quantification result or 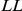 score can be considered “not important” for explaining the observed reads, and can thus be removed from the isoform set when analyzing a particular dataset.

**Algorithm 2:**
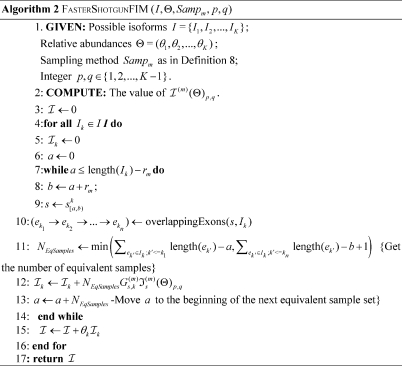
FasterShotgunFIM (*I,*Θ*,Samp_m_,p,q*)

### Use of empirical 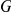 function

To illustrate how non-uniform 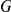 function works, we modeled the bias of RNA-Seq data by aggregating signal of mapped reads along annotated transcripts. A signal map of the first base of mapped reads was generated. The signal was subsequently mapped onto the transcript and aggregated for all genes with signal isoform. An aggregation plot of such a signal map for Young Adult is shown in Text S1. In this aggregation, each transcript is divided evenly into 100 bins, with the signals normalized by the sum of signals across all the bins. The normalized signal at each bin thus represents the probability that a read is generated at certain position of the transcript. These non-uniform probabilities gave more realistic estimation of how the reads are generated, and were plugged into the EM calculations. We compared the quantification results for Young Adult worm with uniform and non-uniform G function. The Pearson correlation score for relative abundance is 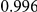, and score for absolute abundance is 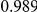. The results are similar for other stages. For the majority of genes, the isoform quantification is largely dependent upon whether reads are compatible with the different isoforms of the gene, while the subtle differences in start position probabilities have little influences on final estimation results. Only for a few genes where the isoform structures are highly similar to each other, the quantification results are different.

Also, there have been some recent works [19], [20] studying the sequencing biases in RNA-Seq data, with more sophisticated modeling utilizing local sequence composition at different positions along the transcript. Based on different assumptions on sequencing bias, their results can be plugged into the 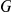 function to derive more realistic quantification results.

### Comparison with existing tools

In order to understand the performance of our method compared with other existing tools, we have conducted additional computational analysis by applying IQSeq and Cufflinks on 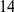 samples from MAQC-3 data [21]. We summarize our result in Figure 12. The genes are categorized by their number of isoforms, and Pearson correlations of the estimated isoform level RPKMs (in logarithmic scale) from the two methods are calculated for each category in each sample. The overall correlation of the isoform quantification results from these two methods is 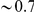 across all samples, which indicates a similar characteristic with a near uniform read-generation assumption. Also, both outputs from IQSeq and Cufflinks have 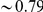 correlation with the Taqman assay [22] (an qRT-PCR technique, which can be considered as a gold-standard here). These results confirm consistency of our method with previous work. Note, however, that with our method one can readily “plug-in” more practical read generation models as illustrated in the previous section, making it a more flexible tool to handle and integrate data from different sequencing technologies. Also, we compared the isoform quantification variances between replicates with the FIM based variance estimations, and their logarithmic values have a correlation of 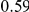 (Figure 3 in Text S1).

**Algorithm 3:**
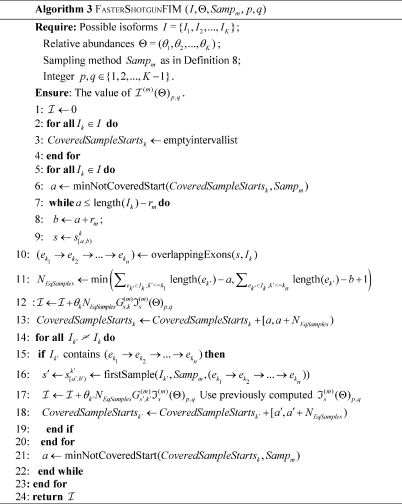
FasterShotgunFIM (*I,*Θ*,Samp_m_,p,q*)

## Discussion

In this paper we explore the problem of integrating different sequencing techniques to quantify the relative abundance of different isoform transcripts, which can be generalized to the problem of estimating the distribution based on partial samples from different sampling techniques. We first introduce a statistical framework to model the generative process of the partial samples, using a “plugin-able” function 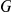 to allow flexible incorporation of different sampling characteristics, and then present the original problem as a maximum likelihood estimation (MLE) problem, with an iterative solution based on expectation maximization, which guarantees a locally optimum answer. This provides a solution to the question of estimating a distribution based on partial samples.

In order to further investigate the problem involving partial samples, we introduce a heuristic based on the Fisher information matrix (FIM) to estimate the variance of the previously presented MLE solution. Also, in order to accelerate the computation of this measurement, we introduce the concept of equivalent partial samples and develop a fast algorithm, Algorithm 3, to accurately calculate FIM, achieving a speedup of 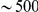 times compared to the brute-force method. Simulation results on both hypothetical and real gene models also show that our FIM-based heuristic gives a good approximation to the value of 
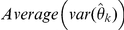
, and accurately predicts the numeric order of this value under different conditions. With this metric, we are also able to demonstrate examples of how to efficiently find low-cost combinations of different sampling techniques to best estimate the isoform compositions in RNA-Seq experiments. Although we are only using individual genes as examples, once we have good assumptions of expression levels of different genes, this procedure can be generalized to all the genes for the low-cost design of actual whole genome RNA-Seq experiments.

What is more, by applying the MLE method to a worm RNA-Seq dataset, we illustrate how we can compare the differential isoform composition between different developmental stages, and how different isoform sets (e.g. with a major/minor isoform missing, with an additional ‘dummy” isoform) will affect the MLE result, especially in terms of the maximized likelihood value, showing that the likelihood score is an effective tool for ranking the “fitness” of isoform sets for a particular gene.

Since IQSeq estimates isoform quantity within a probabilistic framework, it does not directly determine the existence of a certain isoform transcript in the data, but rather gives probability measures (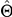) and corresponding RPKM values. The result of a secondary experiment with high precision, e.g. qPCR, on a smaller set of genes, can be used as a gold standard dataset to assist answering such existence questions, with either a simple RPKM value threshold that maximizes the prediction accuracy on the gold standard (training) dataset, or more sophisticated classification techniques that takes multiple characteristics (e.g. 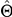, overall gene expression, FIM-based variance estimation) into account.

The FIM-based variance we are trying to estimate in the proposed algorithm focuses mainly on the expected estimation variance based on different read sets of similar on a same sample, and is a measurement of estimation accuracy. In the case of read sets from different biological replicates, the variances of interest there are usually the actual differences in isoform composition of particular genes between/among the replicates, and analysis on such differences can be generally conducted as a downstream procedure after the isoform quantification calculation.

As sequencing technologies constantly evolve, IQSeq will remain able to provide integrated analysis of different datasets with their own sequencing characteristics, and provide guidelines for optimal RNA-Seq experiment design.

## Supporting Information

Text S1 **Supplementary material including additional derivation of formulas, proof of lemmas and theorems, and analysis results.**
(PDF)
