# Supplementary material for: IQSeq: Integrated Isoform Quantification Analysis Based on Next-Generation Sequencing
Source: PLoS One. 2012 Jan 6;7(1):e29175. doi: 10.1371/journal.pone.0029175 (PMC3253133; doi:10.1371/journal.pone.0029175)
Supplement: Text S1 — Supplementary material including additional derivation of formulas, proof of lemmas and theorems, and analysis results. (PDF) [file pone.0029175.s001.pdf]

Text S1. Supplementary Materials for IQSeq: Integrated Isoform Quantification Analysis Based on Next-generation Sequencing

Jiang Du[1], Jing Leng[2], Lukas Habegger[2], Andrea Sboner[3], Drew McDermott[1], Mark Gerstein[1,2,3,*]
1 Department of Computer Science, Yale University, New Haven, Connecticut, United States of America
2 Program in Computational Biology and Bioinformatics, Yale University, New Haven, Connecticut, United States of America
3 Department of Molecular Biophysics and Biochemistry, Yale University, New Haven, Connecticut, United States of America
∗ E-mail: Mark.Gerstein@yale.edu

Supplementary Materials

Maximum Likelihood Estimation

$$\begin{aligned}
\hat{\Theta} &= argmax_{\Theta}\log(Pr(S|\Theta)) && (1)\\
&= argmax_{\Theta}\log\prod_{m=1}^{M}\prod_{i=1}^{N_m} Pr(s_{m,i}|\Theta, Samp_m) && (2)\\
&= argmax_{\Theta}\log\prod_{m=1}^{M}\prod_{s=s_{m,*}} Pr(s|\Theta, Samp_m) && (3)\\
&= argmax_{\Theta}\log\prod_{m=1}^{M}\prod_{s=s_{m,*}}\sum_{k=1}^{K}\delta_{s,k}\theta_k Pr(s|\Theta, Samp_m, I_k) && (4)\\
&= argmax_{\Theta}\log\prod_{m=1}^{M}\prod_{s=s_{m,*}}\sum_{k=1}^{K}\delta_{s,k}\theta_k G_{s,k}^{(m)} && (5)\\
&= argmax_{\Theta}\sum_{m=1}^{M}\sum_{s=s_{m,*}}\log\sum_{k=1}^{K}\delta_{s,k}\theta_k G_{s,k}^{(m)} && (6)
\end{aligned}$$

Expectation Maximization

$$\begin{aligned}
\zeta_{s,k}^{(n)} &= \mathbf{E}_{Z|S,\Theta^{(n)}}\left[Z_{s,k}\right] && (7)\\
&= \mathbf{E}\left[Z_{s,k}|S,\Theta^{(n)}\right] && (8)\\
&= Pr\left(Z_{s,k}=1|s,\Theta^{(n)}\right) && (9)\\
&= \frac{Pr\left(Z_{s,k}=1, s|\Theta^{(n)}\right)}{Pr\left(s|\Theta^{(n)}\right)} && (10)\\
&= \frac{\delta_{s,k}\theta_k^{(n)}G_{s,k}^{(m)}}{\sum_{k'=1}^{K}\delta_{s,k'}\theta_{k'}^{(n)}G_{s,k'}^{(m)}} && (11)
\end{aligned}$$

E step:

$$
\begin{aligned}
Q^{(n)}(\Theta) &= \mathbf{E}_{Z|S,\Theta^{(n)}}\left[\log(Pr(Z,S|\Theta))\right] && (12)\\
&= \mathbf{E}_{Z|S,\Theta^{(n)}}\left[\sum_{m=1}^{M}\sum_{s=s_{m,*}}\log\sum_{k=1}^{K} Z_{s,k}\theta_k G_{s,k}^{(m)}\right] && (13)\\
&= \mathbf{E}_{Z|S,\Theta^{(n)}}\left[\sum_{m=1}^{M}\sum_{s=s_{m,*}}\sum_{k=1}^{K} Z_{s,k}\log\theta_k G_{s,k}^{(m)}\right] && (14)\\
&\quad \text{(for all } Z_{s,*}\text{, one and only one can have a value of 1)} && (15)\\
&= \sum_{m=1}^{M}\sum_{s=s_{m,*}}\sum_{k=1}^{K}\mathbf{E}_{Z|S,\Theta^{(n)}}\left(Z_{s,k}\right)\log\theta_k G_{s,k}^{(m)} && (16)\\
&= \sum_{m=1}^{M}\sum_{s=s_{m,*}}\sum_{k=1}^{K}\zeta_{s,k}^{(n)}(\log\theta_k + \log G_{s,k}^{(m)}) && (17)\\
&= \sum_{m=1}^{M}\sum_{s=s_{m,*}}\sum_{k=1}^{K}\zeta_{s,k}^{(n)}\log\theta_k + C && (18)
\end{aligned}
$$

M step:

$$
\begin{aligned}
\theta_k^{(n+1)} &= \frac{\sum_{m=1}^{M}\sum_{s=s_{m,*}}\zeta_{s,k}^{(n)}}{N} && (19)\\
&= \frac{\sum_{m=1}^{M}\sum_{s=s_{m,*}}\frac{\delta_{s,k}\theta_k^{(n)}G_{s,k}^{(m)}}{\sum_{k'=1}^{K}\delta_{s,k'}\theta_{k'}^{(n)}G_{s,k'}^{(m)}}}{N} && (20)
\end{aligned}
$$

Observed Fisher information matrix

$$
\begin{aligned}
\mathfrak{I}(\Theta)_{p,q} &= -\frac{\partial^2\log(Pr(S|\Theta))}{\partial\theta_p\partial\theta_q}, \text{ where } p,q = 1,...,K-1 && (21)\\
&= \sum_{m=1}^{M}\sum_{s=s_{m,*}} -\frac{\partial^2\log\sum_{k=1}^{K}\delta_{s,k}\theta_k G_{s,k}^{(m)}}{\partial\theta_p\partial\theta_q} && (22)\\
&= \sum_{m=1}^{M}\sum_{s=s_{m,*}} -\frac{\partial^2\left[\log\left(\sum_{k=1}^{K-1}\delta_{s,k}\theta_k G_{s,k}^{(m)} + \delta_{s,K}G_{s,K}^{(m)}(1-\sum_{l=1}^{K-1}\theta_l)\right)\right]}{\partial\theta_p\partial\theta_q} && (23)\\
&= \sum_{m=1}^{M}\sum_{s=s_{m,*}} -\frac{\partial\left[\frac{1}{\sum_{k=1}^{K}\delta_{s,k}\theta_k G_{s,k}^{(m)}}\left(\delta_{s,p}G_{s,p}^{(m)} - \delta_{s,K}G_{s,K}^{(m)}\right)\right]}{\partial\theta_q} && (24)\\
&= \sum_{m=1}^{M}\sum_{s=s_{m,*}} \frac{\left(\delta_{s,p}G_{s,p}^{(m)} - \delta_{s,K}G_{s,K}^{(m)}\right)\left(\delta_{s,q}G_{s,q}^{(m)} - \delta_{s,K}G_{s,K}^{(m)}\right)}{\left[\sum_{k=1}^{K}\delta_{s,k}\theta_k G_{s,k}^{(m)}\right]^2} && (25)
\end{aligned}
$$

Covariance matrix of the maximum likelihood estimator

Let $T(S) = (\hat{\theta}_1, ..., \hat{\theta}_{K-1}, 1 - \sum_{k=1}^{K-1} \hat{\theta}_k)^T$, and $\psi(\Theta) = \mathbf{E}\left[T(S)\right]$. The Cramér-Rao bound states that:

$$cov_\Theta\left(T(S)\right) \geq \frac{\partial \psi(\Theta)}{\partial \Theta}\left[\mathcal{I}(\Theta)\right]^{-1}\left(\frac{\partial \psi(\Theta)}{\partial \Theta}\right)^T \tag{26}$$

, where $\left[\partial \psi(\Theta)/\partial \Theta\right]_{u,v} = \partial \psi_u(\Theta)/\partial \theta_v$, $u = 1, ..., K$; $v = 1, ..., K-1$.
We then estimate $\psi(\Theta)$ by Θ, and use the bound above to estimate the covariance matrix:

$$\frac{\partial \psi_u(\Theta)}{\partial \theta_v} \approx \frac{\partial \theta_u}{\partial \theta_v} \tag{27}$$

$$= \begin{cases} 1 & \text{if } u = v \text{ and } u, v < K; \\ -1 & \text{if } u = K; \\ 0 & \text{otherwise} \end{cases} \tag{28}$$

$$cov_\Theta\left(T(S)\right) \approx \frac{\partial \psi(\Theta)}{\partial \Theta}\left[\mathcal{I}(\Theta)\right]^{-1}\left(\frac{\partial \psi(\Theta)}{\partial \Theta}\right)^T \tag{29}$$

$$= \begin{bmatrix} & \mathbf{I}_{(K-1)\times(K-1)} & \\ -1 & \cdots & -1 \end{bmatrix}_{K\times(K-1)} \times \left(\left[\mathcal{I}(\Theta)\right]^{-1}\right)_{(K-1)\times(K-1)} \tag{30}$$

$$\times \begin{bmatrix} & -1 \\ \mathbf{I}_{(K-1)\times(K-1)} & \vdots \\ & -1 \end{bmatrix}_{(K-1)\times K} \text{, } \mathbf{I} \text{ is the identity matrix} \tag{31}$$

$$= \begin{bmatrix} & \mathcal{I}^{-1}_{(K-1)\times(K-1)} & \\ -\sum_{k=1}^{K-1}\mathcal{I}^{-1}_{k,1} & \cdots & -\sum_{k=1}^{K-1}\mathcal{I}^{-1}_{k,K-1} \end{bmatrix}_{K\times(K-1)} \tag{32}$$

$$\times \begin{bmatrix} & -1 \\ \mathbf{I}_{(K-1)\times(K-1)} & \vdots \\ & -1 \end{bmatrix}_{(K-1)\times K} \tag{33}$$

$$= \begin{bmatrix} & & & -\sum_{k=1}^{K-1}\mathcal{I}^{-1}_{1,k} \\ & \mathcal{I}^{-1}_{(K-1)\times(K-1)} & & \vdots \\ & & & -\sum_{k=1}^{K-1}\mathcal{I}^{-1}_{K-1,k} \\ -\sum_{k=1}^{K-1}\mathcal{I}^{-1}_{k,1} & \cdots & -\sum_{k=1}^{K-1}\mathcal{I}^{-1}_{k,K-1} & \sum_{i=1}^{K-1}\sum_{j=1}^{K-1}\mathcal{I}^{-1}_{i,j} \end{bmatrix}_{K\times K} \tag{34}$$

We also have:

$$\sum_{k=1}^{K} var\left(\hat{\theta}_k\right) = tr\left(cov_\Theta\left(\hat{\Theta}\right)\right) \tag{35}$$

$$= tr\left(cov_\Theta\left(T(S)\right)\right) \tag{36}$$

$$\approx tr\left(\left[\mathcal{I}(\Theta)\right]^{-1}\right) + \sum_{i=1}^{K-1}\sum_{j=1}^{K-1}\left[\mathcal{I}(\Theta)\right]^{-1}_{i,j} \tag{37}$$

This means that we only need $\mathcal{I}(\Theta)$ in order to estimate the performance of our MLE with different sampling method combinations.

Efficient Computation of $\mathcal{I}(\Theta)$

$$\begin{aligned}
\mathcal{I}(\Theta)_{p,q} &= \mathbf{E}\left[\mathfrak{I}(\Theta)_{p,q}\right] && (38)\\
&= \sum_{m=1}^{M}\sum_{s=s_{m,*}} \mathbf{E}\left[-\frac{\partial^2 \log \sum_{k=1}^{K}\delta_{s,k}\theta_k G_{s,k}^{(m)}}{\partial\theta_p\partial\theta_q}\right] && (39)\\
&= \sum_{m=1}^{M} N_m \mathcal{I}^{(m)}(\Theta)_{p,q} && (40)
\end{aligned}$$

where

$$\mathcal{I}^{(m)}(\Theta)_{p,q} = \mathbf{E}_{s\sim Samp_m}\left[-\frac{\partial^2 \log \sum_{k=1}^{K}\delta_{s,k}\theta_k G_{s,k}^{(m)}}{\partial\theta_p\partial\theta_q}\right] \quad (41)$$

is the expected Fisher information matrix of a single partial sample based on $Samp_m$. Thus we need to be able to compute $\mathcal{I}^{(m)}(\Theta)$ in order to obtain $\mathcal{I}(\Theta)$.

$$\begin{aligned}
\mathcal{I}^{(m)}(\Theta)_{p,q} &= \sum_{k=1}^{K}\theta_k \left\{ \sum_{s=s_{[a,b)}^{(k)};\forall[a,b)\in I_k} -G_{s,k}^{(m)} \frac{\partial^2 \log \sum_{k'=1}^{K}\delta_{s,k'}\theta_{k'} G_{s,k'}}{\partial\theta_p\partial\theta_q}\right\} && (42)\\
&= \sum_{k=1}^{K}\theta_k \sum_{s=s_{[a,b)}^{(k)};\forall[a,b)\in I_k} G_{s,k}^{(m)} \mathfrak{I}^{(m)}_{s=s_{[a,b)}^{(k)}}(\Theta)_{p,q} && (43)
\end{aligned}$$

where

$$\begin{aligned}
\mathfrak{I}^{(m)}_{s=s_{[a,b)}^{(k)}}(\Theta)_{p,q} &= -\frac{\partial^2 \log \sum_{k'=1}^{K}\delta_{s,k'}\theta_{k'} G_{s,k'}^{(m)}}{\partial\theta_p\partial\theta_q} && (44)\\
&= \frac{\left(\delta_{s,p}G_{s,p}^{(m)} - \delta_{s,K}G_{s,K}^{(m)}\right)\left(\delta_{s,q}G_{s,q}^{(m)} - \delta_{s,K}G_{s,K}^{(m)}\right)}{\left[\sum_{k'=1}^{K}\delta_{s,k'}\theta_{k'}G_{s,k'}^{(m)}\right]^2} && (45)
\end{aligned}$$

is the Fisher information matrix of a partial sample s from $Samp_m$ at $[a,b)$ in I_k.

Proofs on Equivalent partial samples

$$\begin{aligned}
\mathcal{I}^{(m)}(\Theta)_{p,q} &= \sum_{k=1}^{K}\theta_k \left\{ \sum_{s=s_{[a,b)}^{(k)};\forall[a,b)\in I_k} -G_{s,k}^{(m)} \frac{\partial^2 \log \sum_{k'=1}^{K}\delta_{s,k'}\theta_{k'} G_{s,k'}}{\partial\theta_p\partial\theta_q}\right\} && (46)\\
&= \sum_{k=1}^{K}\theta_k \sum_{s=s_{[a,b)}^{(k)};\forall[a,b)\in I_k} G_{s,k}^{(m)} \mathfrak{I}^{(m)}_{s=s_{[a,b)}^{(k)}}(\Theta)_{p,q} && (47)
\end{aligned}$$

where

$$\mathfrak{I}^{(m)}_{s=s^{(k)}_{[a,b)}}(\Theta)_{p,q} = -\frac{\partial^2 \log \sum_{k'=1}^{K} \delta_{s,k'}\theta_{k'}G^{(m)}_{s,k'}}{\partial\theta_p\partial\theta_q} \quad (48)$$

$$= \frac{\left(\delta_{s,p}G^{(m)}_{s,p} - \delta_{s,K}G^{(m)}_{s,K}\right)\left(\delta_{s,q}G^{(m)}_{s,q} - \delta_{s,K}G^{(m)}_{s,K}\right)}{\left[\sum_{k'=1}^{K} \delta_{s,k'}\theta_{k'}G^{(m)}_{s,k'}\right]^2} \quad (49)$$

is the Fisher information matrix of a partial sample s from $Samp_m$ at $[a,b)$ in I_k.

Definition 1. Two partial samples s_1 and s_2 are equivalent w.r.t. $Samp_m$ if and only if $\mathfrak{I}^{(m)}_{s_1}(\Theta) = \mathfrak{I}^{(m)}_{s_2}(\Theta)$.

Lemma 1. *If $\forall I_k \in I$, $\delta_{s_1,k}G^{(m)}_{s_1,k} = \delta_{s_2,k}G^{(m)}_{s_2,k}$, then s_1 and s_2 are equivalent w.r.t. $Samp_m$.*

Proof. According to Equation 45, we have: $\mathfrak{I}^{(m)}_{s_1}(\Theta) = \mathfrak{I}^{(m)}_{s_2}(\Theta)$. Thus the two partial samples are equivalent w.r.t. $Samp_m$ according to Definition 1. □

Definition 2. A set of partial samples S is an equivalent sample set w.r.t. $Samp_m$ if and only if $\forall s_1, s_2 \in S$, s_1 and s_2 are equivalent w.r.t. $Samp_m$.

Lemma 2. *Given an isoform I_k and a sampling method $Samp_m$, if we divide all its possible partial samples into n non-overlapping equivalent sample sets $S_1, S_2, ..., S_n$, then:*

$$\mathcal{I}^{(m)}(\Theta)_{p,q} = \sum_{k=1}^{K}\theta_k \sum_{i=1}^{n} |S_i| G^{(m)}_{s_i,k}\mathfrak{I}^{(m)}_{s_i}(\Theta)_{p,q}, \textit{ for any } s_i \in S_i \quad (50)$$

Proof. We can rewrite the $\sum_{s=s^{(k)}_{[a,b)};\forall[a,b)\in I_k} G^{(m)}_{s,k}\mathfrak{I}^{(m)}_{s=s^{(k)}_{[a,b)}}(\Theta)_{p,q}$ part in Equation 43 by dividing all the possible $s^{(k,m)}_{a,b}$s into equivalent sample sets $S_1, S_2, ..., S_n$, and then obtain the equation above. □

Definition 3. A simple shotgun sampling method $Samp_m$ generates samples with fixed read length r_m. When sampling from an isoform I_k with length l_k, there are in total $l_k - r_m + 1$ different samples $s^{(k)}_{[a,b)}$, where $a = 0, 1, 2, ..., (l_k - r_m)$; and $b = a + r_m$. Each of these samples has equal probability of being generated from I_k: $G^{(m)}_{s,k} = 1/(l_k - r_m + 1)$.

Lemma 3. *Given the sample generation model $Samp_m$ above, if two samples s_1 and s_2 generated by this method are compatible with the same set of isoforms, i.e. $\delta_{s_1,k} = \delta_{s_2,k}, \forall I_k \in I$, then s_1 and s_2 are equivalent w.r.t. $Samp_m$.*

Proof. If $\delta_{s_1,k} = \delta_{s_2,k} = 0$, then obviously $\delta_{s_1,k}G^{(m)}_{s_1,k} = \delta_{s_2,k}G^{(m)}_{s_2,k} = 0$. Otherwise, if $\delta_{s_1,k} = \delta_{s_2,k} = 1$, then both s_1 and s_2 are partial samples that may be generated by $Samp_m$ from I_k. In this case, according to Definition 3, $G^{(m)}_{s_1,k} = G^{(m)}_{s_2,k} = 1/(l_k - r_m + 1)$. Thus we always have: $\delta_{s_1,k}G^{(m)}_{s_1,k} = \delta_{s_2,k}G^{(m)}_{s_2,k}$, $\forall I_k \in I$. According to Lemma 1, s_1 and s_2 are equivalent w.r.t. $Samp_m$. □

Theorem 1. *Given the sample generation model $Samp_m$ above, if two samples s_1 and s_2 generated by this method overlap with all the junctions in the same set of connected exons $e_{k_1} \to e_{k_2} \to ... \to e_{k_n}$, then s_1 and s_2 are equivalent w.r.t. $Samp_m$.*

Proof. We first prove by contradiction that $\forall I_k \in I$, $\delta_{s_1,k} = \delta_{s_2,k}$:

If $\delta_{s_1,k} \neq \delta_{s_2,k}$, without loss of generality, we assume that $\delta_{s_1,k} = 1$ and $\delta_{s_2,k} = 0$. Then there must exist an exon junction $e_{k_i} \rightarrow e_{k_{i+1}}$, $i \in \{1, 2, ..., n-1\}$, such that $e_{k_i} \rightarrow e_{k_{i+1}}$ is not compatible with I_k (otherwise, as a part of $e_{k_1} \rightarrow e_{k_2} \rightarrow ... \rightarrow e_{k_n}$, s_2 will be compatible with I_k). Since s_1 overlaps with the junction of $e_{k_i} \rightarrow e_{k_{i+1}}$, s_1 is not compatible with I_k either, which will lead to a contradiction to the previous assumption that $\delta_{s_1,k} = 1$. Thus the original statement $\delta_{s_1,k} = \delta_{s_2,k}$ must be true.

Then according to Lemma 3, s_1 and s_2 are equivalent w.r.t. $Samp_m$. □

Use of empirical G function

To illustrate how non-uniform G function works, we modeled the bias of RNA-Seq data by aggregating signal of mapped reads along annotated transcripts. A signal map of the first base of mapped reads was generated. The signal was subsequently mapped onto the transcript and aggregated for all genes with signal isoform. The aggregation plot for Young Adult is shown in Figure S1. Each transcript is divided into 100 bins, accounting for their different lengths. The signals are normalized by the sum of signals in all the bins. The normalized signal at each bin represents the probability that a read is generated at certain position of the transcript. These non-uniform probabilities gave more realistic estimation of how the reads are generated, and were plugged into the EM calculations.

Results with different read generation assumptions

We have also carried out additional analysis comparing the result of IQSeq with uniform read generation assumption and with an empirical read generation model derived from the MAQC-3 dataset [1]. Figure S2 shows that while the overall results are still correlated, there are a significant number of isoforms with different estimated quantity under such different read generation assumptions.

Replicate variance vs. FIM based variance estimation

With the same MAQC-3 data, we also compared the isoform quantification variances between replicates with the FIM based variance estimations, and their logarithmic values have a correlation of 0.59 (Figure S3).

References

1. Bullard JH, Purdom E, Hansen KD, Dudoit S (2010) Evaluation of statistical methods for normalization and differential expression in mrna-seq experiments. BMC Bioinformatics 11: 94.

Figures

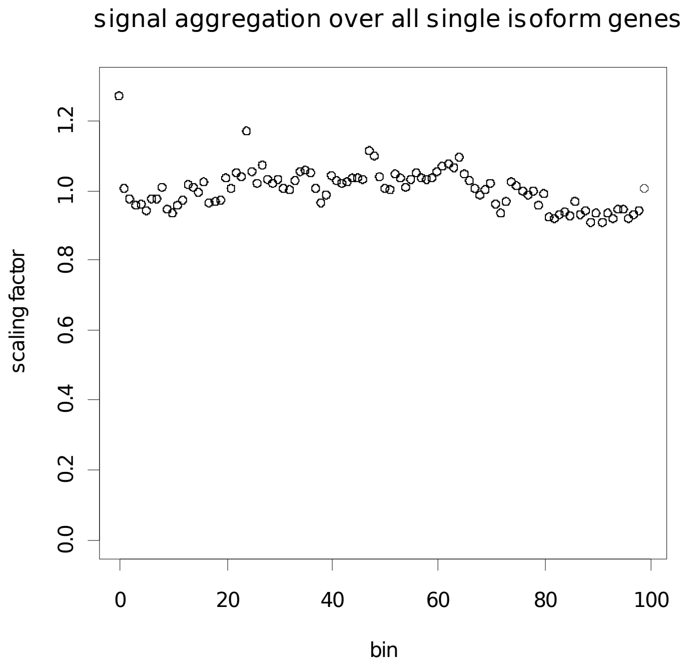

Figure S1. Aggregated signal over all single isoform genes

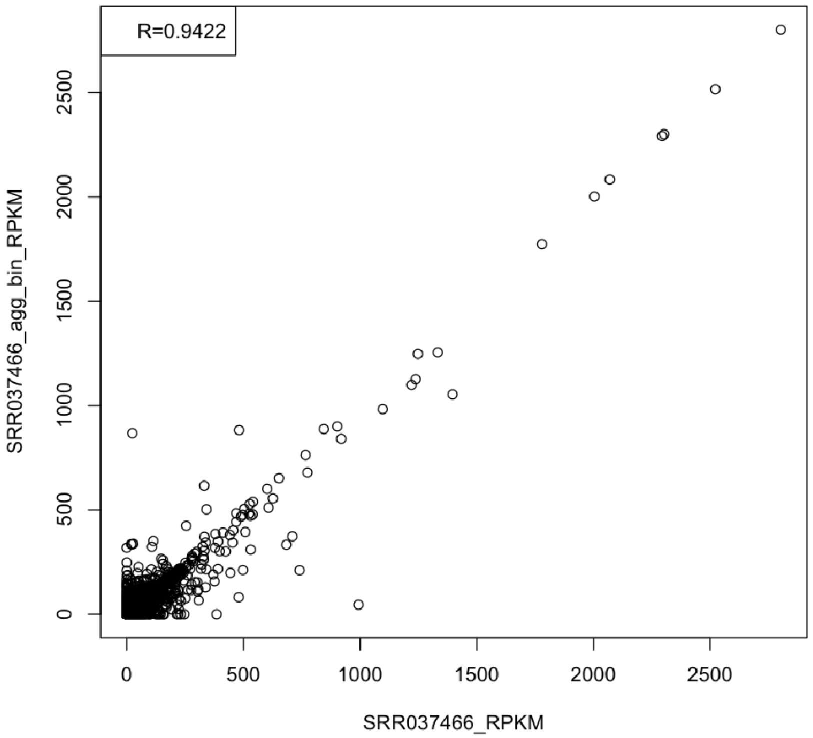

(a) Gene Level RPKM

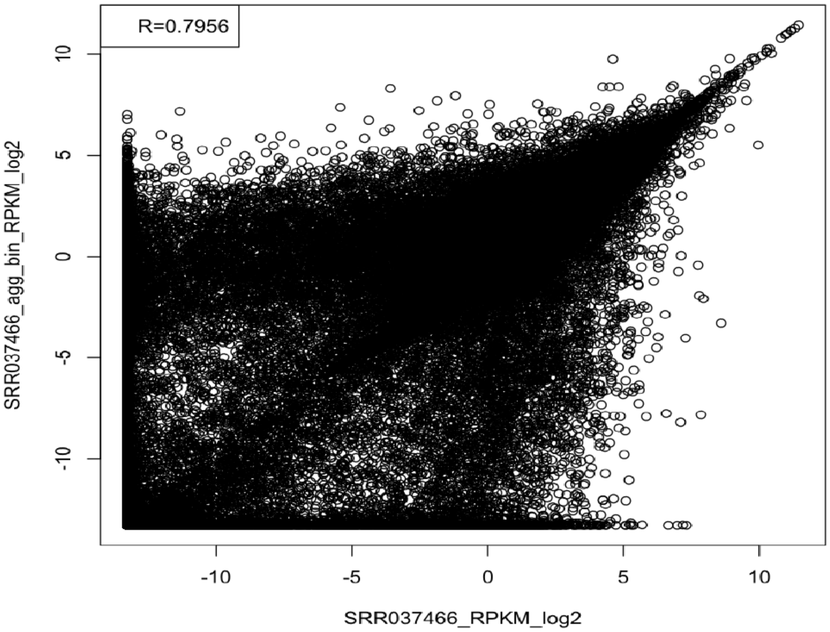

(b) Isoform Level RPKM (log scale)

Figure S2. Results with uniform and non-uniform read generation models

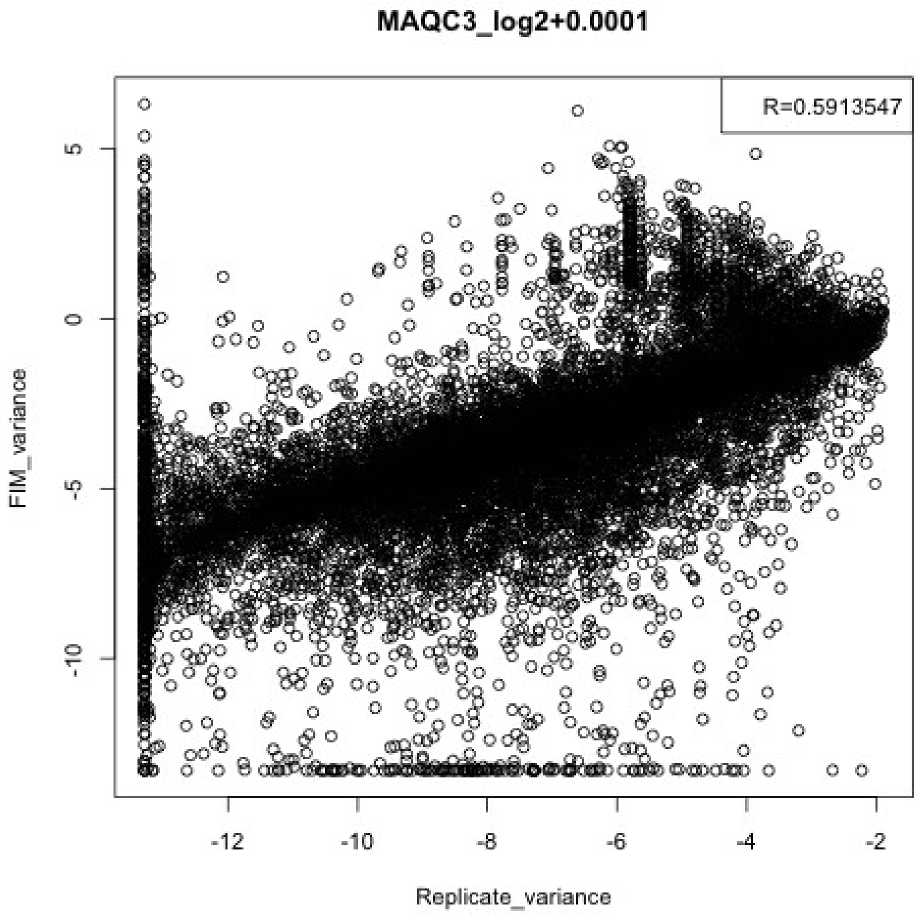

Figure S3. Replicate variance vs. FIM based variance estimation
